# Do Patients with Rheumatoid Arthritis Have an (In)Adequate Level of Physical Activity? A Latent Class Analysis Approach

**DOI:** 10.3390/life14121600

**Published:** 2024-12-04

**Authors:** Sretko Lukovic, Marko Baralic, Nina Tomonjic, Jovana Mihailovic, Aleksandra Neskovic, Marina Vujovic Sestakov, Ivana Pavlovic, Branko Barac, Tatjana Zivanovic Radnic, Predrag Ostojic

**Affiliations:** 1 Institute of Rheumatology, 11000 Belgrade, Serbia; 2 Faculty of Medicine, University of Belgrade, 11000 Belgrade, Serbia; 3 Clinic of Nephrology, University Clinical Centre of Serbia, 11000 Belgrade, Serbia

**Keywords:** rheumatoid arthritis, physical activity, kinesiophobia, sarcopenia, latent class analysis, fatigue

## Abstract

Introduction: Regular physical activity (PA) has a beneficial effect on joint pain, stiffness, strength, flexibility, and aerobic capacity in patients with rheumatoid arthritis (RA). Objective: The aim of this study was to assess the level of PA in patients with rheumatoid arthritis and to identify potential barriers to this activity. Material and Methods: The study involved 132 patients with RA. Participants completed the International Physical Activity Questionnaire (IPAQ), the Functional Assessment of Chronic Illness Therapy—Fatigue Scale (FACIT-F), the Tampa Scale for kinesiophobia (TSK), Strength, Ambulation, Rising from a chair, Stair climbing and history of Falling questionnaire (SARC-F) for sarcopenia assessment, and the Patient Health Questionnaire-9 (PHQ-9) for depression. Basic socio-epidemiological data, disease activity score in 28 joints (DAS_28_), duration of disease, and therapy information were retrieved from electronic patient records. Latent class analysis (LCA) was used to identify subpopulations of patients. Results: The study included 109 women (82.6%) and 23 men (17.4%). Low levels of PA were observed in 16 patients (12%), moderate levels in 70 patients (53%), and high levels in 42 patients (35%). Symptoms of pronounced fatigue were significantly associated with low PA (28.5 ± 11.3 vs. 37 ± 7 vs. 37 ± 10; *p* = 0.002). The risk of sarcopenia was significantly higher in RA patients with low PA (*p* = 0.05). Kinesiophobia was present in all three groups (65.2%). LCA identified two classes. In the first class, patients were more likely to be non-exercisers compared to the second class. Patients in the first class were characterized by a higher probability of being female, obese, with lower education levels. Patients in the first class had pronounced fatigue, kinesiophobia and more frequent symptoms of depression. The second class (65% of the total population) included patients who exercised moderately to frequently (93%) and were middle-aged. They were less obese, highly educated, employed, and majority of them achieved low disease activity or remission. In addition, they had lower risks for sarcopenia, depression, fatigue, and kinesiophobia. Conclusions: This study showed that RA patients with moderate and high levels of PA have better disease control, fewer symptoms of fatigue and depression, and a lower risk of sarcopenia. However, kinesiophobia was significantly present in all three groups, indicating a need for further promotion of this non-pharmacological treatment.

## 1. Introduction

Rheumatoid arthritis (RA) is chronic, systemic, autoimmune disease usually presenting with symmetric polyarthritis of small joints. The extra-articular manifestations of the disease may affect the lungs, kidneys, nerves, skin, or eyes. The WHO estimated that in 2020, about 17.6 million people had rheumatoid arthritis, with higher frequency in women (70%) [1]. People with RA often complain of tenderness and swelling of the small joints, morning stiffness, and reduced joint mobility. In addition, they often report fatigue and symptoms of anxiety and depression. Diagnosis of RA is made in accordance with 2010 ACR/EULAR classification criteria [2]. The pharmacological treatment of RA includes nonsteroidal anti-inflammatory drugs (NSAIDs), corticosteroids, conventional and biological disease-modifying antirheumatic drugs (cDMARDs and bDMARDs), and Janus kinase inhibitors (JAKi) therapy. Non-pharmacological treatment includes lifestyle changes such as diet modification, bodyweight optimization, adequate physical activity (PA), and exercise.

With the introduction of biological therapy in the treatment of RA at the end of last century, the management of RA has been revolutionized. Additionally, the “treat to target” strategy, which aims to achieve complete remission or low disease activity, has made the disease more manageable [3]. However, a substantial number of patients still face unmet needs regarding quality of life (QoL). Many of them have reported issues dealing with chronic pain, social interactions, and problems with emotional health and vitality [4].

The 2018 EULAR recommendations for PA in people with rheumatic diseases, as well as the 2021 EULAR recommendations regarding lifestyle behaviors and work participation to prevent progression of rheumatic and musculoskeletal diseases, emphasized that regular PA and exercising are beneficial for patients with chronic diseases, including RA. Regular PA positively impacts muscle strength and cardiovascular fitness [5,6]. In addition, regular physical activity is recommended as a treatment option for mild and moderate depression often associated with RA [7]. The WHO and American Heart Association (AHR) recommend 150 min per week of moderate aerobic or 75 min moderate-to-vigorous PA (moderate intensity = 3.0 to <6.0 METs (metabolic equivalent of task), vigorous intensity = ≥6 METs, where 1 MET is the rate of energy expenditure at rest). For muscle strength and flexibility, it is recommended to engage in weight lifting or resistance training at least twice a week [8].

Due to chronic illness, lower levels of fitness, pain, biomechanical abnormalities, and the fear of exacerbating their arthritis through exercise, many patients avoid regular PA. The primary goal of this study was to investigate the level of PA in adults with RA. The secondary goals were to identify the most prevalent barriers to regular PA and to explore specific demographic, clinical, and behavioral differences between patients with varying levels of PA.

## 2. Materials and Methods

This was an observational study approved by the local Ethics Committee (18/42) in May 2023. The study included 132 patients with RA that were treated at the Institute of Rheumatology Belgrade. The RA diagnosis was made according to ACR/EULAR criteria from 2010 and with a minimum of six months of disease duration [6]. The exclusion criteria were heart failure with restricted and preserved EF (HFrEF and HFpEF), respiratory insufficiency requiring oxygen therapy, and active malignancy. The study did not include neuromuscular or psychiatric disorders as exclusion criteria, but patients were asked for comorbidities, and there were no reported neuromuscular or psychiatric disorders. Patients were invited to participate in the study during regular ambulatory visits. Written consent was given by 170 patients, and 78% of them completed the survey. Basic demographic, epidemiological, and disease-related data were obtained from patient electronic charts.

Disease activity was assessed using the Disease Activity Score in 28 joints (DAS_28_), which includes information about swollen and tender joints (28 joints evaluated), acute-phase reactants sedimentation (SE) or C-reactive protein (CRP), and visual analog scale (VAS) of the patient’s general function. A score above 5.1 indicates very active disease, while the score below 2.6 indicates remission. The score between 3.2 and 2.6 reflects low disease activity, and the score between 3.2 and 5.1 is indicative for moderately active RA [9].

### 2.1. Patient-Reported Outcomes Measures (PROMs)

Participants filled out the short version of the International Physical Activity Questionnaire (IPAQ), which contained nine questions regarding different levels of PA. Patients were asked to answer how many minutes they spent in low, moderate, or vigorous PA or being sedentary. The results were expressed in MET minutes per week (continuous) and as low, moderate, and high PA (categorical variables). One MET is the amount of energy expended at rest (about 3.5 mL O_2_/kg/min or 1 Kcal/kg/h), representing basal metabolism [10]. It is estimated that energy expenditure during low PA (e.g., regular walking) is 3.3 times higher than at rest, 4 times higher in moderate PA (e.g., jogging, riding a bicycle), and 8 times higher in vigorous PA (sprinting, doing high-intensity training).

To calculate the weekly PA, the investigator multiplied the minutes spent on each type of PA by the corresponding energy expenditure constant and the number of days the activity was performed, summing the results. Scoring a high level on IPAQ means the participant engaged in a minimum of 3000 MET minutes per week, equating to about an hour or more every day in moderate and vigorous PA. Moderate level on IPAQ means achieving a minimum of 600 MET minutes per week in PA (about half an hour daily). Scores below 600 MET minutes per week were classified as low PA [11].

To assess the presence of fatigue, we used the Functional Assessment of Chronic Illness Therapy (FACIT) Fatigue Scale, a short 13-item questionnaire that is easy to answer about the level of fatigue in daily life during the last seven days. The level of fatigue is measured on four-point Likert scale (4 = not at all fatigued to 0 = very much fatigued) and the score under 30 indicates serious fatigue [12].

Kinesiophobia is defined as irrational and debilitating fear of physical activity or movement resulting from a feeling of vulnerability to painful injury or re-injury. The Tampa Scale of Kinesiophobia (TSK) is a 17-item questionnaire, also rated on a four-point Likert scale, with statements that have been later linked to the model of fear-avoidance, fear of work-related activities, fear of movement, and fear of re-injury. The score above 37 indicates kinesiophobia [13,14]. The Strength, Assistance in walking, Rise from chair, Climb stairs, and Fall questionnaire (SARC-F) is commonly used in screening for sarcopenia. The questionnaire consists of five simple questions regarding difficulties in everyday activities like rising from a chair, climbing the stairs, using assistance in walking, and the frequency of falls during the last year. It has a maximum of ten points, and a score above 4 is associated with the risk of sarcopenia presence [15]. To assess the symptoms of depression Patient Health Questionnaire 9 (PHQ-9) was used, a reliable tool containing nine questions about specific symptoms experienced over the past 2 weeks. Responses were rated on a 4-point Likert-type scale (ranging from 0—not at all to 3—nearly every day) where a score above 15 indicates moderately severe depression [16].

### 2.2. Latent Class Analysis

Latent class analysis (LCA) is a statistical method mostly used in behavioral sciences to investigate population heterogeneity. By using categorical variables, LCA identifies subgroups (latent classes) with similar characteristics within the population. It is a person-oriented mixture model that groups individuals based on their patterns of responses to observed variables. Based on statistical theory, individual scores on a set of variables are driven by their latent class membership [17]. The SPSS version 28 software (IBM, Armonk, NY, USA) and EZR statistical programs were used for data analysis [18]. For continuous variables, ANOVA was performed to detect significant differences between groups. For categorical variables, chi-squared test was performed. The “poLCA” package was installed in the EZR version 1.61 program to conduct LCA.

## 3. Results

Of the 170 RA patients initially enrolled, 132 completed the study (78% response rate). The study cohort consisted primarily of women (82.3%) with a median age of 43 (IQR: 24–61) and 17.4% of men with a median age of 44 (IQR: 24–61) years. Most of the patients had a high school (48.5%) or faculty degree (50%), and regarding employment status, about 25% of them were unemployed. The medical charts review detected that hypertension (HTN) was observed with moderate frequency in the patients (29/132, 22%), followed by osteoporosis (5/132, 12.9%). Notably, almost half of our patients did not have other recorded comorbidities, but overweight and obesity related to calculated BMI were present in 58/132 (43.9%). All included patients undergo steroid treatment at one time of disease evolution. In addition, we had data on cumulative years of corticosteroid use for each patient, but there was no association with the specific level of PA. Almost one third of the patients were smokers (30.3%) (Table 1).

A low level of PA was present in 16/132 (12%) patients, a moderate level of PA in 70/132 (53%) patients, and a high level of PA was detected in 46/132 (35%) patients. DAS_28_ in the group with low PA (3) was statistically significantly higher than in the group with moderate PA (2.9) or high PA (2.5) (*p* = 0.036). There were no statistically significant differences between groups with different level of PA in terms of their age, obesity, disease and treatment duration, and osteoporosis. However, the state of being overweight and obesity were notable in all three groups, primarily in the group with the moderate PA (48.6%). Most of the patients in these three groups were treated with biologic therapy or JAKi, but without significance comparing PA levels (62.5% vs. 64.3% vs. 56.5%, *p* = 0.699). There was a significant association between higher fatigue scores and low PA levels (*p* = 0.002). The kinesiophobia was notably present in every observed group with the highest score in group with low PA (40), but without statistical significance (*p* = 0.641). Patients with low PA had significant chance of having sarcopenia in opposite to patients with moderate or high PA (4 vs. 2.2 vs. 2, *p* = 0.006). The symptoms of depression were significantly associated with low PA (*p* = 0.023) (Table 2).

**Figure 1:**
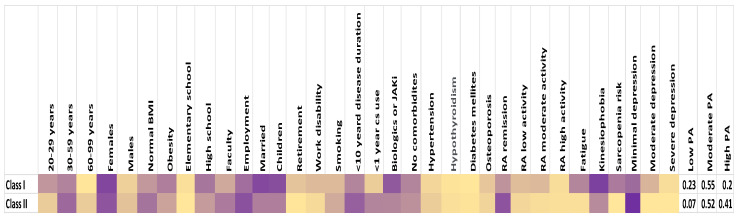
LCA identified two latent classes. Color gradient indicates membership probability for every variable in each class—from lowest (beige) to highest (violet). CS—corticosteroids, JAKi—Janus kinase inhibitors, PA—Physical activity.

The LCA identified two latent classes with 0.39 and 0.61 population shares, respectively. The members of the first class had a higher probability of having low PA than members in the second class (23% vs. 7%). Nine out of ten members in first class were females, middle-aged, with dominantly modest education (the probability of having a high school degree is 62%). Comparing classes, the chance of being employed was lower in this class, opposite to the probability of being married and having kids. The members of first class had more possibility to have work disability or be retired. Clinically, members of the first class had a 31% chance of having active disease, a 46% chance that the disease lasted longer than ten years, and a higher chance of using corticosteroids over the years. The fatigue was much more present in the first class compared with the second class (54% vs. 4%). The member of the first class had more chance of having kinesiophobia than members in the second class (91% vs. 51%). The member of this class had a 33% chance of having symptoms of moderate to severe depression. Members of the second class had a 41% chance of having high PA compared to a 20% chance in the first class. The probability of being male in the second class was 20%, and the average age of members was 30–59 years (69%). The chance of being overweight or having obesity was lower than in the first group (37% vs. 57%). The chance of being highly educated was higher in this class (59% vs. 34%) as well as positive employment status. Members in the second class had higher chances of being smokers and having HTN. The chance of disease remission or low disease activity was higher in this group (89%). The member in this class had a lower chance of having osteoporosis (10% vs. 20%). In the second class, the risk of sarcopenia was completely absent, whereas it was 60% in the first class. The second-class member had minimal chance of having moderate or severe symptoms of depression. The LCA model had good fit statistics, with a Bayesian Information Criterion (BIC) of 3380 and an entropy score of 0.82, suggesting clear classification of patients into latent classes (Figure 1).

## 4. Discussion

This was one observational study on PA levels in patients with RA where 12.5% of patients had low PA, 53% of patients had moderate PA, and 34.5% of them had high PA. These results are similar to results from Van Den Berg et al., who reported that about 58% of patients with RA followed their recommendation for adequate PA [19]. In addition, one cross-sectional study of the general population of the UK showed that low levels of PA were more prevalent in those with RA (23%) compared with controls (15%) [20]. The results in our study implicate the need for a more objective method for investigating the level of PA. Also, there is a lack of data on how many patients had a low level of PA before RA onset. One Swedish study of 617 patients reported that only 8% of inactive patients were inactive before disease onset, which could be beneficial for physicians to intervene earlier with promotion of regular PA [21].

RA is characterized by chronic inflammation resulting from complex interactions between immune cells and inflammatory cytokines, predominantly interleukin-1 (IL-1), interleukin-6 (IL-6) and tumor necrosis factor-alpha (TNFα). Regular PA promotes anti-inflammatory response in the body. During the exercising there is overexpression of IL-6; however, unlike inflammation, TNFα and IL-1 are suppressed. This muscle-mediated IL-6 overexpression stimulates hepatic lipolysis and glycogenolysis, supplying more glucose to the muscles opposite to the macrophage -mediated IL-6, which stimulates acute inflammation and production of CRP [22]. Patients in our group with low PA had higher DAS_28_ compared with patients with moderate or high PA. The level of CRP, part of this complex parameter, was higher in patients with low PA. These results are similar to the results of a meta-analysis performed by Brady et al., who found the positive effect of PA on disease activity, functional ability, and fatigue [23]., There were no significant differences in the use of biologics or JAKi between groups with different levels of PA. Despite the presence of this kind of therapy in every group, there were unmet needs of patients, indicating a space for PA as a additional therapeutic tool.

Regular PA, due to its beneficial effects, can reduce increased cardiovascular (CV) risk in RA patients. Studies have demonstrated that regular and frequent PA positively affects peripheral vascular resistance, endothelial function, and baroreceptor sensitivity, leading to better control of hypertension. Exercise enhances the translocation of insulin-mediated glucose transporter type 4 to the sarcolemma, resulting in increased glucose uptake by the cells for up to 24 h. Over time, this can help prevent or reduce insulin resistance, which is common in overweight and obese patients [22]. A study by Fernanda et al. showed that obesity is highly prevalent in patients with RA and associated with other CV factors [24]. Our study found that overweight and obesity were present in each reported group, indicating the need for further regular evaluation of CV risks in patients and implementation of regular PA.

RA is a chronic systemic disease that leads to joint inflammation and can result in inflammatory and chronic neuropathic pain. Patients with RA often experience symptoms of depression and sleeping disturbances. In our study, patients with low levels of PA had higher PHQ-9, indicating a greater risk for depression compared to those with moderate or high PA. A meta-analysis conducted in the United States with nearly 2500 participants and 500 reviewed citations showed that regular PA reduced depressive symptoms in adults with adult rheumatic conditions, including RA [25]. Fatigue is another common symptom in RA patients. This study found that fatigue was present in one-fifth of patients and was significantly more common in those with low PA compared to those with moderate or high PA and resonated with a previous study. Katz et al. identified physical inactivity as independent risk factor for having fatigue [26]. Interestingly, although fatigue is a barrier to PA, studies have shown that regular PA can reduce fatigue. Katz et al. also showed that simple physical activity, such as step-targeted walking with pedometers, optimally reduced fatigue and pain and improved Health Assessment Questionnaire (HAQ) scores in RA patients. It is crucial that healthcare professionals inform patients about the benefits of regular PA.

Patients with RA experience recurrent disease flares leading to decreased activity due to fear of movement and the potential worsening of symptoms [27]. In our group, kinesiophobia was present in almost two-thirds of the group, similar to findings by Öztürk et al., where kinesiophobia was reported in 70% of patients. The pathological fear of movement, coupled with low PA, increases the risk of obesity, hypertension, mood disorders, and sleep disturbances. Öztürk et al. also have shown that kinesiophobia is associated with fear of falling, depression, and a lower quality of life, affecting social, emotional, physical, and mental functions [28]. Patients with RA often experience a loss of muscle mass and strength due to chronic inflammation, TNFα overexpression (known as rheumatoid cachexia), joint destruction, obesity, physical inactivity, and age. The relationship between sarcopenia and disease severity in autoimmune diseases is still debated. In patients with systemic sclerosis and sarcopenia, higher scores of skin thickening, disease severity, and activity with respect to non-muscle-depleted patients were observed. Our observational study found an association between low PA and the risk of sarcopenia presence [29,30]. The presence of sarcopenia, combined with kinesiophobia and fatigue, increases the risk of falls, fractures, and cumulative mortality. Resistance training or weight lifting twice a week for about 30 min, alongside adequate nutrition, is recommended as the first-line treatment to mitigate sarcopenia in RA patients [31].

LCA identified two latent classes with different PA levels. This analysis is commonly used in behavioral science to identify groups with similar characteristics in large, heterogenous populations. Interestingly, the first class, characterized by a higher likelihood of physical inactivity, included patients with lower education levels, work disabilities, or those who were retired, highlighting the social and economic factors influencing participation in PA. The prototype of the patient from this latent class is middle-aged woman, has children, but reports symptoms of depression, fatigue, and kinesiophobia. Her DAS_28_ score indicates low to moderate disease activity and her RA has lasted more than 10 years. Finding an optimal way to encourage regular PA for this middle-aged woman with modest education and likely low income is challenging. Rheumatologists should inform patients about the benefits of being physically active and the potential consequences of inactivity. Additionally, more education is essential for patients, rheumatologists, and physiotherapists in this area. Finally, improving the social and economic circumstances of patients could facilitate regular and adequate PA.

## 5. Conclusions

This study showed that higher levels of physical activity were associated with better disease outcomes, including lower disease activity (DAS_28_), reduced fatigue, and lower prevalence of depressive symptoms. However, kinesiophobia emerged as significant factor influencing PA levels indicating a serious need for further education of patients and healthcare professionals in this domain. Additionally, patients with higher level of PA could be mentors to the smaller groups of patients with low PA level giving them support in challenging barriers and subsequently improving their PA level. We used LCA, commonly used in behavioral sciences, but rarely in rheumatology, trying to find “hidden” similarities in heterogenous RA population. In terms of PA, LCA emphasized that the role of social environment of patients is important as much as clinical features of patients. 

Despite its strengths, this study has some limitations. The cross-sectional design limits our ability to infer causal relationships between PA, disease activity, and mental health. Longitudinal studies are needed to establish the directionality of these associations. Regarding sarcopenia, the study team has plan to investigate the presence of sarcopenia and effect of regular PA using more objective methods. Additionally, the use of self-reported measures for PA (IPAQ) and depressive symptoms (PHQ-9) may introduce reporting bias, though these tools are widely validated in clinical research.

## Figures and Tables

**Table 1:** Basic demographic, epidemiological, and clinical information.

N = 132 (100%)	N, (%)
Sex	
Male	23 (17.4)
Female	109 (82.6)
Age (years)	43.5 ± 11
60+ years	9 (6.8)
Education	
Elementary school	2 (1.5)
High school	64 (48.5)
Faculty	66 (50)
Employment	101 (76.5)
Marital status	90 (68.2)
Children	88 (66.7)
Retirement	10 (7.6)
Working disability (confirmed by NHIF)	17 (12.6)
RA activity	
Remission	91 (68.9)
Low	18 (13.6)
Moderate	16 (7.1)
High	7 (6.3)
Comorbidities	
Hypertension	29 (22)
Diabetes mellites	2 (1)
Osteoporosis	17 (12.9)
Hypothyroidism	5 (3)
No comorbidities	65 (49)
Overweight and obesity	58 (43.9)
Smoking	40 (30.3)
IPAQ (MET minutes per week)	2601 ± 1912
Fatigue	28 (21.2)
Kinesiophobia	85 (65.2)
Risk for sarcopenia	27 (20.5)

NHIF—National Health Insurance Fund, IPAQ—International Physical Activity Questionnaire, MET—Metabolic Equivalent of Task.

**Table 2:** Characteristics of patients with different levels of PA.

	Low Level of PA, N = 16 (100%)	Moderate Level of PA, N = 70 (100%)	High Level of PA, N = 46 (100%)	*p*
MET minutes per week	359 ± 106	1742 ± 648	4688 ± 1614	0.001
Age (years)	46.3 ± 9.5	42 ± 12	42.3 ± 10	0.307
60+ years	2 (12.5)	5 (7.1)	2 (4.3)	0.531
BMI	24.9 ± 6	25.1 ± 4.8	24.5 ± 5	0.856
Overweight and obesity	5 (31.1)	34 (48.6)	19 (41.3)	0.410
Osteoporosis	4 (25)	7 (10)	6 (13)	0.271
DAS_28_	3 ± 1.3	2.9 ± 1	2.5 ± 1.3	0.036
Disease duration (years)	8.2 ± 5	10.8 ± 8	9.7 ± 8	0.504
Treatment duration (years)	7 ± 4.4	9.9 ± 7–8	8.6 ± 8	0.499
Biologics or JAKi	10 (62.5)	45 (64.3)	26 (56.5)	0.699
Duration of biologics or JAKi (years)	2.72 ± 3.1	3.22 ± 4.2	2.3 ± 3.7	0.460
Functional status (HAQ)	0.6 ± 0.5	0.65 ± 1	0.8 ± 1.2	0.694
FACIT Fatigue	28.5 ± 11.3	37.7 ± 7	37 ± 10	0.002
TSK	40 ± 8	38 ± 6.4	38.4 ± 5.8	0.641
SARC-F	4 ± 2.2	2.2 ± 1.9	2 ± 2	0.006
PHQ-9	7.6 ± 5	4.9 ± 4.6	4 ± 3.9	0.023

Low level of physical activity (PA) = 0–600 MET minutes per week, Moderate level of physical activity (PA) = 600–3000 MET minutes per week, High level of physical activity (PA) = more than 3000 MET minutes per week. MET—Metabolic equivalent of task. BMI—Body Mass Index, DAS_28_—Disease Activity Score (A score below 2.6 indicates remission while the score above 5.1 indicates very active disease. The score between 3.2 and 2.6 indicates low disease activity and score between 3.2 and 5.1 is indicative for moderately active RA). JAKi—Janus kinase inhibitors, HAQ—Health Assessment Questionnaire, FACIT fatigue scale (score lower than 30 indicates serious fatigue), TSK—Tampa scala of kinesiophobia (score higher than 37 predicts kinesiophobia), SARC-F (score higher than 4 is risk for having sarcopenia), PHQ9—Patient Health Questionnaire for depression (score 0–4 None or minimal, 5–9 Mild, 10–14 Moderate, 15–19 Moderately severe depression).

## Data Availability

The original contributions presented in this study are included in the article. Further inquiries can be directed to the corresponding author.
